# Human Emotion Experiences Can Be Predicted on Theoretical Grounds: Evidence from Verbal Labeling

**DOI:** 10.1371/journal.pone.0058166

**Published:** 2013-03-06

**Authors:** Klaus R. Scherer, Ben Meuleman

**Affiliations:** Swiss Center for Affective Sciences, University of Geneva, Geneva, Switzerland; National University of Singapore, Singapore

## Abstract

In an effort to demonstrate that the verbal labeling of emotional experiences obeys lawful principles, we tested the feasibility of using an expert system called the Geneva Emotion Analyst (GEA), which generates predictions based on an appraisal theory of emotion. Several thousand respondents participated in an Internet survey that applied GEA to self-reported emotion experiences. Users recalled appraisals of emotion-eliciting events and labeled the experienced emotion with one or two words, generating a massive data set on realistic, intense emotions in everyday life. For a final sample of 5969 respondents we show that GEA achieves a high degree of predictive accuracy by matching a user’s appraisal input to one of 13 theoretically predefined emotion prototypes. The first prediction was correct in 51% of the cases and the overall diagnosis was considered as at least partially correct or appropriate in more than 90% of all cases. These results support a component process model that encourages focused, hypothesis-guided research on elicitation and differentiation, memory storage and retrieval, and categorization and labeling of emotion episodes. We discuss the implications of these results for the study of emotion terms in natural language semantics.

## Introduction

Emotions have often been considered as defying a systematic, analytical approach to the underlying mental and somatic states. However, recent decades have seen an exponential growth of publications on emotion in many different disciplines: A search for the keyword “emotion” on Web of Science within the social sciences showed that, between 1990 and 2011, the number of publications on this subject has annually multiplied by a factor 1.13. This is all the more surprising since the term *emotion* has been widely used only since the beginning of the 19th century, replacing terms such as passion, affection, or sentiment [1]. Recently, in parallel with the growth in scientific interest, the everyday popularity of the term has also increased enormously. Emotions have become desirable, as evidenced by the many types of products that–according to the advertisements–promise to deliver intense emotional experiences. These changes may have modified the somewhat negative connotation of the original term in the sense of *bodily*, *involuntary,* and *irrational* [2], sometimes generating the implicit assumption that emotions cannot be scientifically studied in the sense of lawful causation. This tendency has been reinforced by the influential claim of William James [3] that emotions are nothing but the proprioception of our bodily symptoms, a claim that is currently being revived in the form of various constructionist accounts of emotion [4], [5].

Yet, in recent years psychological and neuroscience research has generated a massive amount of empirical data on different mechanisms in emotion generation and regulation [6]. At the same time, there has been a “somatization” of emotion research, with the bulk of it being directed at physiological correlates (see Part 2 of [7]), facial and vocal expression [8], and, more recently, the underlying neural circuitry (see Part 1 of [7]). Given the extraordinary complexity and variety of emotional responses across individuals and situations, however, few efforts have been made to predict the nature of *specific* emotional episodes occurring in real life. Not surprisingly, this leads to a fundamental question: Can the *qualities* of human emotion experiences be scientifically predicted at all? Or can they be approached only through post hoc phenomenological analysis of self-report on subjective experience?

In trying to answer this question, one first has to define the phenomenon. What do we mean by emotion? In recent years, there has been increasing convergence on a definition of emotion as a componential process that involves (*a*) different levels of cognition (situation appraisal); (*b*) motivational changes (action tendencies); (*c*) physiological reactions; (*d*) motor expression; and (*e*) subjective feeling [9]. Appraisal theory places special emphasis on the cognitive component and assumes that changes in the other four components are largely driven (in the sense of recursive causality) by the appraisal process, which encompasses evaluations of relevance, goal congruence, coping potential, and norm compatibility (see reviews in [10]). This framework allows the generation of specific hypotheses regarding the effects of appraisal on component patterning [11]–[13].

Concretely, the first author’s Component Process Model (CPM) of emotion [13], [14] claims that the outcome of each appraisal check changes the state of all other emotion components (which represent subsystems of the organism) and that the changes produced by the result of a preceding check are modified by that of a consequent check. The sequential appraisal of a personally relevant event on a generic set of criteria is expected to produce (*a*) changes in the support system (e.g., heart rate decrease, skin conductance increase); (*b*) changes in the motivation (or action tendency) system (e.g., focusing the sensory perception areas toward a novel stimulus); (*c*) changes in goal priority assignment in the executive subsystem (e.g., attempting to deal with a potential emergency); and (*d*) changes in alertness and attention in an overall monitoring subsystem (feeling). This architecture allows the development of very specific hypotheses with respect to the predicted changes in different emotion components as a consequence of specific appraisal results (see Table 5.3 in [14], pp 109–112). Many of the predictions of the CPM concerning the physiological changes, motor expression, and even brain activity have been tested and confirmed in the laboratory by experimentally manipulating the appraisal checks [13].

The preceding paragraph describes low-level effects of appraisal on individual emotion components. On a more molar level, many appraisal theories have predicted specific emotion categories–identified by emotion words–as a function of specific appraisal profiles (see Figure 19.1 in [11], for a comparative overview). An example based on the CPM is shown in Table 1 for selected emotions. Here, the focus is on the subjective feeling component (the conscious reflection of the changes in all components as described earlier) and the categorization and labeling of the emotion experience with the help of an emotion word or expression. Appraisal theorists have conducted a large number of empirical studies, including field studies [15], [16], to test their theoretical predictions, and the results lend strong support to the theoretical framework [11], [13]. However, in each of these studies, only a small part of the complete set of predictions can be tested, as the experimental manipulations are generally restricted to a subset of emotions. Moreover, it is often difficult to assess the underlying appraisal checks in an experimental setting.

**Table 1:** Predicted appraisal profile for selected emotions.

Appraisal check	Joy	Rage	Fear	Sadness
**Relevance**				
**Novelty**				
Suddenness	High	High	High	Low
Familiarity	Open	Low	Low	Low
Predictability	Low	Low	Low	Open
**Intrinsic pleasantness**	Open	Open	Low	Open
**Goal/need relevance**	High	High	High	High
**Implication**				
Cause: agent	Open	Other	Other	Open
Cause: motive	Open	Intentional	Open	Chance
Outcome probability	Very high	Very high	High	Very high
Discrepancy fromexpectation	Open	Dissonant	Dissonant	Open
Conduciveness	Conducive	Obstructive	Obstructive	Obstructive
Urgency	Low	High	Very high	Low
**Coping potential**				
Control	Open	High	Open	Very low
Power	Open	High	Very low	Very low
Adjustment	Medium	High	Low	Medium
**Normative significance**				
Internal standards	Open	Open	Open	Open
External standards	Open	Low	Open	Open

Adapted from [14].

To address this issue, Scherer [17] suggested using an expert system approach to test the complete set of CPM predictions. Concretely, the question is whether it is possible to predict the emotion words that people will use to describe their emotional experiences on the basis of self-reported appraisal. The Geneva Expert System on Emotions (GENESE) was the first system developed for this purpose [17]. GENESE used simple distance assessment algorithms to determine the relative similarity between a user’s input vector (of recalled appraisals for a specific event) and prototypical category vectors representing the knowledge base. The knowledge base consisted of a set of prototypical emotion vectors with numerical representations for the type of predictions shown in Table 1. GENESE contained prototypical vectors for 14 emotions on 15 appraisal checks. Users were asked to recall a situation in which they had experienced a strong emotion and to answer 15 questions designed to assess the different appraisal checks. Comparing the user’s input vector to the 14 prototype vectors, the system presented a “diagnosis” of the experienced emotion on the basis of the closest fit (with some appraisal criteria being given greater weight on theoretical grounds), and the user was asked whether this was correct. If the answer was “no,” a second diagnosis was presented based on the second closest fit. For 231 situations entered by different participants, the overall percentage of hits was 77.9% (180 first and second hits compared with 51 true misses).

Although this first study provided a general demonstration of the feasibility of the approach, there were a number of limitations, in particular the fact that users might have shown an acquiescence tendency in accepting a diagnosis in cases in which they had no clear preconception on how they would have labeled the emotional experience. To remedy this problem, we developed a new system called the Geneva Emotion Analyst (GEA), programmed in PHP and presented as a freely accessible Web experiment in which users have to possibility to label the reported emotion situation *before* the GEA diagnosis is communicated. The program requires users to briefly describe a situation that caused an emotion and to choose one or two labels from a list of 13 common emotions that, to the user’s mind, best characterized the experience. The option of indicating two labels was provided based on the frequent finding that realistic emotions in everyday life are often mixed [15], [18], [19]. In addition, as compared with the earlier study, the number of appraisal variables was expanded from 15 to 25; a more extensive weighting system for appraisal criteria was used; and users were asked to choose appropriate emotion labels for their experience before entering the information on their appraisals, rather than after the diagnosis (to avoid an influence of the latter on labeling). These changes make the present version of the GEA much more appropriate to the collection and analysis of complex emotion episodes. Importantly, the new system was tested on an enlarged sample of participants of close to 6000 subjects, including men and women of three different language groups, thus significantly enhancing the generalizability of our results.

## Results

### Emotion Combinations

**Figure 1:**
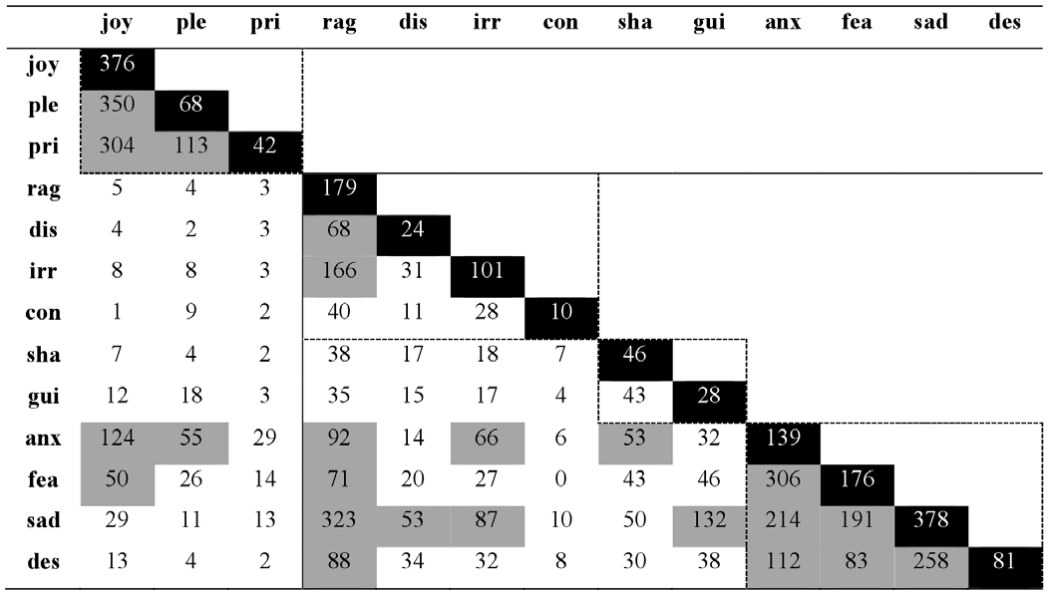
Cross-tabulation of emotion combinations. Black diagonal cells tabulate users who chose only one emotion label. Gray cells indicate combination frequencies larger than 50. Dotted boxes delineate emotion families, in order: happiness, anger, guilt, and distress. joy, joy; ple, pleasure; pri, pride; rag, rage; dis, disgust; irr, irritation; con, contempt; sha, shame; gui, guilt; anx, anxiety; fea, fear; sad, sadness; des, despair.

A cross-tabulation of the users’ first and second emotion choices (see Figure 1) showed that most users provided two labels rather than one. Only 27.6% of users thought a single label was sufficient to describe the emotion they experienced. This finding is consistent with earlier research showing that people often require more than one label to accurately describe their emotional state [15], [18], [19], and it supports our decision to expand the number of choices for this study. Log-linear analysis of the cross-tabulation table across gender or language group (English, French, German) further indicated that the combination of choices did not vary across these groups, with χ^2^(156) = 131, *p* = 0.928, for gender, and χ^2^(312) = 338, *p* = 0.149, for language groups, respectively.

For 13 emotions, there are 78 potential emotion combinations (disregarding order of choice). With the exception of fear–contempt, all possible combinations occurred at least once in the data set. This finding is particularly striking for combinations that, intuitively, might appear to be mutually exclusive, such as pride–shame. Furthermore, many mixed states occurred more frequently in the data set than pure states. For instance, pride occurred more frequently in combination with joy and pleasure than it did alone. These findings support the notion that pure emotions are the exception rather than the rule. An emotion may be complex in any number of ways, representing blended feelings, sequential feelings, or even a highly specific meaning not covered by existing labels. Finally, to exclude the possibility that participants chose combinations at random, a test of quasi-independence was applied to the cross-tabulation of choices. Results revealed that choice combination deviated significantly from being random, χ^2^(131) = 3689.731, *p*<0.0001. In other words, there was a systematic relation between first-choice emotion and second-choice emotion (Figure 1).

### Family Structure

In general, most of the emotion combinations represented natural families of emotion such as the *happiness* family (joy, pleasure, and pride), the *anger* family (rage, disgust, contempt, and irritation), the *shame/guilt* family, and the *distress* family (anxiety, fear, sadness, and despair). Less frequently, we observed combinations between positive and negative emotions. If they did occur, they most often involved the negative emotions of anxiety or fear. An inspection of the situation descriptions for joy-fear choices, for instance, revealed that such combinations were often related to dramatic life events such as the birth of a first child. Similarly, when persons engage in high-risk activities such as gambling or extreme sports (e.g., skydiving), they can simultaneously feel fear and joy.

It should be noted, finally, that the nature of an emotion combination is likely to differ in the within and across family cases. In the former, respondents may have checked two terms because they were not quite sure which one might be the best to use (uncertainty), or to qualify the special nature of the situation (refinement). In the across case, the degree of blending would be expected to be much greater. However, in quite a number of these cases one might also expect the different emotions to occur sequentially in the emotion episode, such as a good outcome in an anxiety producing situation giving rise to joy, or an angry first response to a frustration leading to consequent sadness.

### Predictive Accuracy

Table 2 shows the prediction results for 5969 users, with rows representing the number of emotions correctly predicted by the GEA system and columns representing the extent of user agreement with the predicted labels. In 51% of the cases, the *first* prediction matched the label(s) provided by the user (in 7% with a complete match–if only one label was given–and in 44% with a partial match–if two labels were given). A binomial test indicated that this percentage was much higher than chance at 2 random guesses out of 13, χ^2^(1) = 5812, *p*<0.0001. In those cases in which only the second prediction showed a match with the input label(s), 17% of the users thought it came close to what they felt. In only 10% of the cases did users think that the diagnosis was completely wrong. Log-linear analysis of predictive accuracy across gender and language (English, French, German) groups indicated that accuracy did not vary across these groups, with χ^2^(9) = 2.2, *p* = 0.987, for gender, and χ^2^(18) = 8.7, *p* = 0.966, for language groups, respectively.

**Table 2:** Cross-tabulation of the Geneva Emotion Analyst (GEA) prediction accuracy against the user’s evaluation of the GEA prediction (in absolute numbers and percentages).

	User evaluation of GEA prediction				
	Wrong	Partial	Close	Match	Total
**Prediction accuracy**					
** None**	542	1201	898	0	2641
	9%	20%	15%	0%	44%
** Secondary match**	48	122	113	0	283
	1%	2%	2%	0%	6%
** Primary match**	0	0	0	2653	2653
	0%	0%	0%	44%	44%
** Two matches**	0	0	0	392	392
	0%	0%	0%	7%	7%
**Total**	590	1323	1011	3045	5969
	10%	22%	17%	51%	100%

A primary match indicates the closest prototype matched either of the user’s two labels; a secondary match indicates the second closest prototype matched either of the two labels.

Because the analysis of choice cross-tabulation had suggested the presence of emotion families in the data, we also analyzed predictive accuracy of the GEA system with respect to these families. Table 3 presents a confusion matrix of GEA accuracy by emotion family, affording more insight into why prediction is successful. Even when the GEA is mistaken about the exact label, it predicts the emotion family well above chance at 58.6%, χ^2^(1) = 3596.394, *p*<0.0001 (against 1 random guess out of 4).

**Table 3:** Confusion matrix of the Geneva Emotion Analyst (GEA) predicted emotion families against the user’s chosen emotion family (in absolute numbers).

	GEA prediction			
	Happiness	Anger	Shame/guilt	Distress
**User choice**				
** Happiness**	1066	108	21	297
** Anger**	49	474	10	740
** Shame/guilt**	16	124	23	309
** Distress**	228	515	53	1936
** Class hit rate**	71.4%	37.2%	4.9%	70.9%

### Theory Versus Data

We next examined the extent of agreement between our theoretical prototypes and the empirical emotion centroids in the data (for the complete profiles, see *Supporting Information*, Table S1 in File S1). The results are summarized in Table 4, indicating fairly high agreement between theory and data. Eight out of 13 correlations proved to be significant. The strongest correlations were obtained for contempt and pride, *r* = .75 and *r* = .74, respectively. The weakest correlations occurred for sadness and anxiety, *r* = .33 and *r* = .38, respectively, suggesting that the theoretical appraisal profiles for these emotions need substantial revision to improve the predictive accuracy.

**Table 4:** Pearson correlation coefficients between theoretically predicted emotion prototypes, the empirical emotion centroids obtained from the GEA data reported here, and empirically obtained semantic profiles for the respective emotion terms (GRID).

Emotion	Theo-GEA	Theo-GRID	GEA-GRID
Sadness	0.33	0.52	0.58**
Joy	0.69**	0.58*	0.62**
Rage	0.70***	0.68**	0.77***
Anxiety	0.38	0.39	0.59**
Fear	0.62**	0.57*	0.46*
Irritation	0.46*	0.50	0.69**
Shame	0.39	0.17	0.72***
Contempt	0.75***	0.68**	0.78***
Guilt	0.44	0.43	0.47*
Disgust	0.41	0.47	0.68**
Pleasure	0.60*	0.61*	0.66**
Despair	0.55*	0.58*	0.59**
Pride	0.74***	0.55	0.62**
Mean	0.54	0.52	0.63

Theo Theoretical predictions, GEA feature profile data generated by the GEA system and first reported in this article, GRID feature profile data generated in the GRID study on semantic profiles of emotion terms [23]; *P<0.05, **P<0.01, ***P<0.001.

Overall, however, it appeared that the agreement between theory and data was quite good (*r*_mean_ = 0.54), especially considering that the correlations in Table 4 currently neglect second-choice emotion information. The empirical centroid of joy, for instance, represented an aggregation of many different emotion combinations containing joy (see Method section), whereas the theoretical prototype represents a pure state of joy. Thus, the empirical centroids must be considered as somewhat noisy estimates of the pure states in the data.

## Discussion

Our results show that we are able to predict (or, strictly speaking, postdict) with only one guess the emotion labels that close to 6000 participants used to describe an enormously variable set of emotion episodes, with a precise match in 7% of the cases and a partial match in 44% of the cases (if two labels were provided by the respondent). Even in those cases in which our prediction did not match either label, it was sufficiently close to the user’s categorization of the situation such that in only 10% of all cases did the user declare the diagnosis returned by the GEA system to be erroneous (Table 2). The accuracy of the system could partly be explained by successful prediction of emotion family: even when the GEA system could not produce an exact label match, it could still correctly identify the emotion family far above chance level (58% overall family accuracy). This finding corresponded to the observation that participants also tended to choose within-family combinations for mixed emotions.

### Limitations

The current study has some obvious limitations. Participants were self-selected, and we have no information concerning the conditions under which they completed their GEA session. It is possible that some individuals used the system several times or that they did not sufficiently understand the instructions. Thus, even after eliminating 65 dubious cases, some invalid or noisy data points may still be left among the almost 6000 runs. We therefore stress that our results should be taken to be conservative estimates of the GEA’s performance. Nevertheless, entering the data on an emotion episode is time-consuming; all those who continued the survey until completion were most likely quite motivated to seriously test the system. Most descriptions entered should therefore be of reasonable to high quality. In addition, the size of the data set should render the most important trends visible for analysis, regardless of any impurities in the data. The advantage of our procedure is that we have obtained a large sample of real-life emotion episodes that are of sufficient intensity to bring out the unique character of specific emotions or emotion blends, something that is difficult to obtain in controlled research designs. It is likely that the excellent prediction results of the GEA system are at least in part due to the intense, realistic nature of the reported situations.

The most important limitation is that the cross-sectional self-report nature of the data does not allow drawing firm conclusions about the causal role of appraisal on emotion elicitation and differentiation. Essentially, we studied participants’ memory representation of important events in their life with respect to the verbal labeling of the emotional experience, and the recalled or reconstructed appraisals of the event. It might also be that the nature of the task encouraged participants to choose more prototypical, and thus more easily predictable, emotions (together with the motivation of being a “good test case”). On the other hand, the fact that many participants used two labels for better characterization seems to contradict that assumption.

It should also be noted that the selection of the list of emotion labels is likely to have affected the results. For example, embarrassment, gratitude, jealousy, envy and many other emotions are not included, and rage and irritation are included in place of the more commonly used term “anger”. There are therefore questions about the coverage of the expert system outside the range of sampled emotions. It is also true that this selectivity may have reduced the accuracy of predictions because participants may have recalled an embarrassment incident (for example) but not been able to label it as such because the word did not appear in the list of available options. This might also help to explain the frequency of mixed emotions that is reported here.

Despite these limitations, the results of our study seem to be consistent with two fundamental assumptions made by appraisal theorists: (I) Emotional experiences produced by specific events are stored in memory as unique bounded episodes that can be differentiated by specific appraisal configurations and (II) the semantic meaning of emotion used to label such experiences contains representations of these appraisal configurations that are sufficiently stable to allow reliable coding, memory storage and retrieval, and social communication. Below we examine these two aspects.

### The Role of Appraisal

As to (I), over the last three decades, a large number of appraisal studies have focused on self-report and labeling, largely supporting the predictions of appraisal theory [10], [19], [20]. We believe that our results can be interpreted as supporting the plausibility of the claim that the configuration of appraisal results (based on a small number of theoretically derived generic criteria) is an important factor in the elicitation and differentiation of emotion episodes and their subsequent labeling [11], [21]. The data show that the cognitive component of an emotion episode, mainly the appraisal process, seems to be sufficient to differentiate the set of major modal emotions. These results demonstrate that the theoretically derived predictions, upon which most appraisal theories converge, constitute a solid framework for the further analysis of the causal mechanisms in emotion generation and differentiation.

This should not be misunderstood as cognitive imperialism, neglecting other factors that affect the feeling component and the categorization and verbalization of emotions. As described earlier, the CPM assigns a major role to the central representation of proprioceptive feedback from motivational changes such as action tendencies and the related bodily changes and motor expressions. The central assumption is, however, that most of these changes are *driven by the appraisal results* and are thus likely to support, at least in large part, the labeling suggested by the appraisal configuration. We suggest that the differentiation of the major categories of modal emotions is directly based on the prototypical appraisal configuration but that more fine-grained distinctions, for instance, between the members of an emotion family (e.g., anger, rage, irritation, frustration, spite, annoyance), will depend on other factors, such as proprioceptive feedback of bodily manifestations. In addition to these intra-organismic determinants, the verbal labeling of emotional experiences can also be strongly influenced by situational constraints or strategic considerations, just as nonverbal expressions can (*push* vs. *pull* factors; see [8], pp 423–424).

Our results also support the claim that emotion differentiation is an emergent process, depending entirely on the (nonlinear) interaction of the appraisal checks and thus capable of producing an extremely large number of highly differentiated states, of which only some modal clusters correspond to established categories and labels. The fact that most respondents provided two labels to describe the experience suggests that frequently a single label did not exhaustively characterize the experience (although in many cases one can imagine that the two labels describe different moments in the entire emotion episode linked to a particular event; e.g., relief after escaping a dangerous situation).

### Semantics of Emotion Words

As to (II), the CPM predicts that appraisal generates an almost unlimited variety of emotion outcomes and accompanying feeling components. In consequence, any categorization is necessarily fuzzy and verbal labels are probably assigned on the basis of prototype matching [22], [5]. Clearly, the prediction vectors used in this study correspond to such prototypes. Labels for graded emotion concepts (e.g., within emotion families–anger, rage, irritation) may be needed to label subcategories. Most likely, the use of linguistic labels or expressions to describe the conscious part of feeling rarely covers the complete conscious experience (due to the lack of appropriate verbal concepts or strategic communication intentions). At the same time, the implications of the chosen verbal description may go beyond the content of the emotional experience, as the denotation and connotation of the concepts used in the verbalization may add surplus meaning.

Further work in this area should address both (*a*) the process of becoming conscious and consequently categorizing and labeling emotion episodes on the basis of appraisal results, motivational adjustments, somatovisceral symptoms, and motor expression on the one hand, and (*b*) individual, contextual, and cultural factors on the other. We suggest to view the act of labeling as an act of *reference*. This assumes that in order to allow for effective communication, the meaning of most words reliably captures essential facts about the world and human behavior. Concretely, this means that the labeling of emotion episodes consists of matching features of the integrated feeling experience with the semantic profiles of emotion words in everyday language. Scherer and collaborators [23], [24] have developed a semantic grid methodology (GRID) to identify such semantic profiles for major emotion words. The results of a massive study of 24 such words in 25 languages in more than 30 countries shows that emotion words in all of these languages can be mapped well onto a set of major features representing the different components. Most importantly, the data show that the appraisal part of the profile does indeed seem to be the central factor driving differentiation [24]. We believe that this is an important bit of evidence, given that the causality of appraisal in actually occurring emotion processes is difficult to establish experimentally (however, see work by Roseman and colleagues for a recent example, [25]). The plausibility of our assumption that appraisal results drive other emotion components is further increased if we can demonstrate conclusively that appraisal configurations outperform features from other emotion components in differentiating prototypical emotion categories and labels.

The GRID study tested the extent to which 24 major emotion terms can be classified on the basis of profiles of 142 features representing all of the emotion components (appraisal, bodily reactions, expression, action tendencies, and feeling). In an overall multiple discriminant analysis, these 142 features allowed classification of the 24 emotions with a cross-validated hit rate of 82.1%. If only the 31 features representing the appraisal component were used, a cross-validated hit rate of 70.7% was reached, only about 10% less than for the combined discriminative power of all component features. Successively adding other components in a series of multiple discriminant analyses shows that adding action tendencies adds about 5% to the hit rate, consequently adding bodily reactions and expression adds another 5%, and finally adding feelings adds the remaining 2% [24]. This suggests that the appraisal feature profiles alone allow the lion’s share of the variance to be explained in the semantic differentiation of the emotion terms. This result is highly consistent with the claim of appraisal theories, and especially the CPM, that the appraisal results causally drive the changes in the other components and produce a level of synchronization or coherence that is constitutive for the occurrence of an emotion episode.

It has been suggested [13] that it is precisely the degree of synchronization that might determine whether a bounded period of time following an event is being considered as a coherent emotional experience, is stored as a retrievable unit in memory, and is available for recall. An important question for further research is exactly which elements representing the different components are being stored in memory, how these elements are interconnected, and what cues serve as markers for recall. For example, are nonverbal categories or verbal labels stored as part of the package, facilitating recall, or is the storage essentially phenomenal, subject to categorization and labeling upon recall whenever this is required for communicative purposes? The connectedness of elements from different components of emotion have been highlighted by early theories of emotional memory that proposed semantic networks [26] or associative networks [27], possibly organized on different levels of processing [28]. The relationship between the synchronization of components during the microgenetic unfolding of the emotion process on the one hand and the semantic profiles of emotion words used in labeling as well as the interconnectedness of multicomponential elements during memory storage and recall on the other, constitute important issues for future research in the affective neurosciences.

Given the important role of the semantics of emotion terms in the storage and recall of emotional experiences, it is instructive to examine how well the appraisal features represented in the semantic profiles of the emotion terms used in this study (as measured in the GRID study) can predict our empirically obtained centroid vectors. The Pearson correlations between the corresponding vectors are shown in column 3 of Table 4. Judging from these strong and highly significant correlations, the appraisal profiles reported by the participants for their respective emotional experiences correspond very well indeed to the prototypical semantic profiles of the corresponding labels.

One could argue that participants have simply retrieved an experience from memory on the basis of the label. This is incorrect as participants freely chose an emotional experience to test GEA and freely described the situation in detail *before* choosing a label in a list. One could further argue that once they had chosen a label they responded to the appraisal questions in terms of the prototypical semantic profiles rather than the recalled or reconstructed appraisals they experienced in the situation. This alternative explanation cannot be ruled out on the basis of the present design. However, it seems unlikely to hold as they had already recalled the situation in a detailed fashion to provide the free description. Furthermore, if they had been guided by the semantics of the chosen term, it is difficult to understand why 72.4% of the participants chose a second term to describe the nature of their experience more precisely. This is strong evidence against the argument that the responses might be based on the semantics of the words rather than the real experiences as recalled from memory. Finally, it is instructive that the correlations between the theoretically predicted profiles and the GRID profiles (see Table 4, column 2) are lower than those between the theoretical prediction and the GEA data profiles. If participants had simply responded in terms of semantic prototypes, the opposite should be the case.

In conclusion, given that the results reported in this article cannot be reasonably explained without accepting that the two fundamental assumptions outlined earlier are likely to be valid, we suggest that adopting a componential appraisal model may well allow us to finally envisage an exact science of emotion. This is not to deny the important role of proprioceptive feedback from the body, individual differences, contextual factors or sociocultural determinants (the latter being of particular importance given the resurgence of the universality-relativity debate [29], [30]). These need to be investigated more extensively and systematically and added into our explanatory models as determinants or moderator variables. Similarly, we need to refine our model to account for, and possibly predict cases of typical mixed emotions, such as sadness and guilt (see Table 1). But they should not serve as an excuse to abandon the nomothetic investigation of the emotion process. Emotion research is in urgent need of principled, theory-guided research in which concrete hypotheses on the causation and the temporal unfolding of emotion are being experimentally tested.

## Method

### Sample

Within the period of study, 6034 users submitted data to the online GEA system, 65 of which were removed from the final analysis due to missing data (4 observations) or response bias (61 observations). Response profiles were considered biased when only two or fewer unique response values were used throughout the questionnaire, or when over 70% of responses were of the “not applicable” kind. This reduction left a sample of 5969 users (3982 females, 1987 males). The age of the users ranged from 12 to over 60, with most aged between 20 and 40 years (approximately 60%). Three language groups were represented in the data, English speaking (2780), French speaking (2595), and German speaking (594). Participants were self-selected, as the GEA system was (and still is) freely available on the website of the Swiss Center for Affective Sciences (www.affective-sciences.org/emotion_analyst). Data were gathered during the course of several years.

### GEA System

The procedure and format of the system largely corresponded to the one used in [17]. After choosing one of three language options (English, French, or German), the user recalls and describes an emotion episode and labels it with one or two emotion labels from a list of 13, including pride, joy, pleasure, rage, irritation, contempt, disgust, guilt, shame, anxiety, fear, sadness, or despair. These emotions were drawn from [14] and are considered to be common emotions. The GEA system then poses a series of 34 questions, 25 of which represent theoretically specified appraisal variables (*Supporting Information*, Table S2 in File S1). The remaining questions deal with contextual information that is not currently used in the prediction. Each appraisal variable is measured on a 5-point scale assessing to what extent the appraisal was or was not present during the emotion episode, ranging from “1 =  not at all” to “3 =  moderately” to ”5 =  extremely,” with “0 =  not applicable.”

The resulting vector of 25 appraisal answers is then processed by the GEA algorithms using weighted prototype matching. The architecture of this system is straightforward. Its core consists of a 13×25 matrix containing 13 prototype vectors (*Supporting Information*, Table S1 in File S1). Each row of this matrix corresponds to one of the 13 modal emotions and each column corresponds to one of 25 appraisal variables. For a particular emotion, the value that each appraisal variable takes reflects the “expected” value that this appraisal should take as predicted by theory [14]. Hence, these theoretical prototypes can be thought of as centroids or cluster centers in a 25-dimensional appraisal space.

Emotion prediction proceeds in three steps. First, the user’s input vector is weighted according to a set of 25 predefined weights. These weights reflect the relative importance that should be attached to appraisal variables in discriminating between emotions, such that appraisals with large weights will dominate the solution of the prediction system. Second, appraisal variables with a theoretical prediction value of zero are omitted from the final distance matching vectors. This is because the theory makes no explicit prediction on these values, and hence they are considered as missing values. Third, the distance from the remaining weighted input vector to each of the 13 prototypes is computed by using Manhattan distance (i.e., the sum of absolute differences). The two prototypes with the shortest distance to the input vector are then chosen as the system’s predictions. The method of matching observed data to a theoretical prototype by Manhattan distance is graphically depicted in Fig. S1 in File S1. Note that the system also computes Euclidean and Penrose distance, but these are currently unused. Here, Manhattan distance was chosen because of its robustness against outliers for high-dimensional data.

Finally, the GEA system returns the predicted emotion labels to the user. If a match is found between the closest prototype and either of the two emotion labels provided by the user (primary match), the prediction is considered correct and the user is notified of this success. If a match is found between the second closest prototype and either of the two emotion labels provided by the user (secondary match), or if no match is found, both prototypes are returned to the user with the suggestion that the participant may have experienced a mixed state of emotions. The user is then asked to indicate whether this proposal is (*a*) completely wrong, (*b*) covers at least part of what was felt, (*c*) comes close to what has been felt, or (*d*) is completely correct.

### Data Analysis

Data analysis proceeded in three steps. In a first step, we analyzed the cross-tabulation of emotion choices to determine whether the combination of choices varied across gender and language group, and whether combinations were systematic or random. This we tested using log-linear analysis for contingency tables. For the question on systematicity, a special case of log-linear analysis was applied called the test of quasi-independence, which tests whether the off-diagonal elements in a square agreement table deviate significantly from random association [31]. In a second step, the proportion of successful predictions of the GEA system was compared to chance level using a binomial test, and compared across gender and language groups using log-linear analysis. In the third step, we examined the extent of agreement between the theoretical GEA emotion profiles, the empirical GEA emotion profiles, and the semantic GRID emotion profiles (*Supporting Information*, Table S1 in File S1). The GEA and GRID appraisal profiles were obtained by aggregating appraisal values across the 13 relevant emotions. For the GEA data, these emotions corresponded to the first-choice emotion categories (Note that using the second choice as a grouping variable did not substantially alter the correlation presented in Table 4.). For the GRID data, only the English, French, and German language cases of the full dataset were used, so as to maximally conform to the sample characteristics of the GEA data. The three types of profiles were then compared for each emotion by calculating all pair-wise Pearson’s correlation coefficients (Table 4). To ensure compatibility with the prediction system, theoretical zeroes were treated as missing values when calculating correlations.

## Supporting Information

File S1 **Supporting Information.** Figure S1, Prototype matching with Manhattan distance for two-dimensional artificial appraisal data. User-observed appraisal input is depicted as data clouds in the 5 by 5 appraisal space. Theoretical emotion centroids for joy (circle), rage (diamond) and fear (square) are situated in this appraisal space. For a given user’s appraisal input (black dot), the GEA system calculates the Manhattan distance to each emotion centroid. The emotion with the shortest distance is outputted as the GEA’s prediction, in this case fear. Table S1, Standardized appraisal profiles for the 13 Geneva Emotion Analyst emotions. Displayed are the theoretically predicted profiles (THEO), the empirically found profiles for the GEA data (GEA), and the empirically found profiles for the GRID data (GRID). Pearson correlations between each pair of columns are displayed on the bottom row. Empty cells indicate that the corresponding appraisal item was not present for the respective profile. Refer to Table S2 for appraisal variable names. Table S2, List of appraisal questions in the GEA questionnaire, including appraisal check name, data abbreviation, and weight used in the adjustment of the distance functions.
(DOCX)
