# Supplementary material for: Human Emotion Experiences Can Be Predicted on Theoretical Grounds: Evidence from Verbal Labeling
Source: PLoS One. 2013 Mar 6;8(3):e58166. doi: 10.1371/journal.pone.0058166 (PMC3590138; doi:10.1371/journal.pone.0058166)

Table S1

|  | **Emotion** | | | | | | | | | | | | | | | | | | | | | | | |
| --- | --- | --- | --- | --- | --- | --- | --- | --- | --- | --- | --- | --- | --- | --- | --- | --- | --- | --- | --- | --- | --- | --- | --- | --- |
|  | **Sadness** | | | | **Joy** | | | | **Rage** | | | | **Anxiety** | | | | **Fear** | | | | **Irritation** | | | |
| **Appraisal** | **THEO** | **GEA** | | **GRID** | **THEO** | **GEA** | | **GRID** | **THEO** | **GEA** | | **GRID** | **THEO** | **GEA** | | **GRID** | **THEO** | **GEA** | | **GRID** | **THEO** | **GEA** | | **GRID** |
| **Pleas** |  | -0.52 | | -0.71 |  | 1.36 | | 1.40 |  | -0.59 | | -0.88 |  | -0.17 | | -0.72 | -0.48 | -0.33 | | -0.75 |  | -0.53 | | -0.70 |
| **Unpleas** |  | 0.49 | | 0.77 |  | -1.22 | | -1.63 |  | 0.64 | | 0.78 |  | 0.19 | | 0.44 |  | 0.37 | | 0.55 |  | 0.34 | | 0.78 |
| **Sudden** | -0.48 | 0.15 | | 0.10 | 0.18 | -0.31 | | 1.42 | 0.84 | 0.31 | | 0.67 | -0.48 | 0.00 | | -0.70 | 0.84 | 0.16 | | 0.95 | -0.48 | 0.02 | | 0.00 |
| **Predict** | -0.48 | -0.06 | | 0.12 | -0.48 | 0.08 | | 0.77 | -0.48 | -0.10 | | 0.08 | 0.18 | 0.00 | | 0.02 | -1.14 | -0.18 | | 1.16 | 0.18 | -0.07 | | 0.03 |
| **Famil** | -0.48 | -0.06 | | -0.02 | -0.48 | 0.04 | | 0.86 | -0.48 | -0.06 | | -0.35 |  | -0.11 | | -0.50 | -1.14 | -0.14 | | -1.04 | -1.14 | 0.3 | | -0.47 |
| **Impconse** | 0.84 | 0.17 | | -0.47 | 0.84 | 0.00 | | 1.46 | 0.84 | 0.01 | | -0.77 | 0.84 | 0.12 | | -0.07 | 1.50 | 0.16 | | -0.57 | 0.18 | -0.39 | | -0.68 |
| **Moralacc** |  | -0.23 | |  | 0.84 | 0.48 | |  | -0.48 | -0.33 | |  |  | -0.02 | |  |  | -0.26 | |  | 0.18 | -0.22 | |  |
| **Violnorm** |  | 0.08 | | 0.00 | -1.14 | -0.40 | | -1.28 | 0.84 | 0.33 | | 0.98 |  | 0.00 | | 0.46 |  | 0.04 | | 0.46 | 0.18 | 0.29 | | 0.76 |
| **Chance** | 0.84 | -0.06 | | 0.04 | 0.84 | 0.10 | | 1.15 | -1.14 | -0.09 | | -0.67 | 0.18 | 0.04 | | -0.35 | 0.84 | 0.23 | | -0.08 | -1.14 | -0.28 | | -0.21 |
| **Ownbehav** |  | -0.10 | | -0.49 |  | 0.47 | | 1.00 |  | -0.33 | | -0.14 | -1.14 | 0.05 | | -0.22 | -1.14 | -0.20 | | -1.46 |  | -0.24 | | -0.59 |
| **Intent** | -1.14 | -0.25 | |  |  | 0.66 | |  | -1.14 | -0.33 | |  | -1.14 | -0.04 | |  | -1.14 | -0.21 | |  | -1.14 | -0.32 | |  |
| **Consimag** |  | -0.20 | | 0.26 | 0.84 | 0.53 | | -1.63 |  | -0.12 | | 0.89 |  | -0.12 | | 0.45 |  | -0.22 | | 0.21 |  | -0.13 | | 1.02 |
| **Othbehav** |  | 0.06 | | 0.14 | 0.84 | 0.07 | | 0.08 | 1.50 | 0.16 | | 0.92 | 0.18 | -0.05 | | -0.50 | 0.84 | -0.17 | | -0.05 | 1.50 | 0.3 | | 0.28 |
| **Othint** | -1.14 | -0.01 | | -0.50 | 1.50 | 0.17 | | 1.36 | 1.50 | 0.17 | | 0.90 | 0.18 | -0.06 | | -1.33 | 0.84 | -0.27 | | -0.86 | 0.84 | 0.22 | | -0.39 |
| **Consfelt** | 1.50 | -0.06 | |  | 1.50 | 0.03 | |  | 1.50 | 0.02 | |  | -1.14 | 0.04 | |  | -1.14 | -0.20 | |  | 1.50 | 0.01 | |  |
| **Consexp** | 0.18 | -0.14 | | 0.03 | -0.48 | 0.11 | | 1.05 | -0.48 | -0.10 | | -0.36 |  | -0.01 | | -0.68 | -1.14 | -0.12 | | -1.04 |  | -0.06 | | -0.49 |
| **Conearfu** | -1.14 | -0.12 | |  | 0.84 | 0.13 | |  | 0.18 | -0.15 | |  | -0.48 | -0.02 | |  | 0.84 | -0.09 | |  | -1.14 | 0.09 | |  |
| **Cofarfu** | -1.14 | -0.05 | |  | -1.14 | 0.04 | |  | -1.14 | 0.02 | |  | 0.84 | -0.05 | |  | -0.48 | 0.07 | |  | -1.14 | 0.02 | |  |
| **Posoutc** | -1.14 | -0.37 | | -0.70 | 1.50 | 1.03 | | 1.43 | -1.14 | -0.52 | | -0.96 | -1.14 | -0.12 | | -0.62 | -1.14 | -0.18 | | -0.94 | -1.14 | -0.39 | | -0.69 |
| **Negoutc** | 0.84 | 0.38 | | 0.88 | -1.14 | -0.90 | | -1.57 | 1.50 | 0.51 | | 0.85 | 0.84 | 0.25 | | 0.65 | 1.50 | 0.21 | | 0.76 | 0.84 | 0.27 | | 0.85 |
| **Unjust** |  | 0.37 | | 0.69 | -1.14 | -0.69 | | -1.54 | 0.84 | 0.66 | | 1.11 |  | 0.06 | | 0.28 |  | -0.01 | | 0.22 | 0.84 | 0.43 | | 1.12 |
| **Avoidabl** | -1.14 | 0.17 | | -0.42 |  | -0.56 | | -0.55 | 0.84 | 0.38 | | 0.69 |  | 0.08 | | -0.28 |  | 0.04 | | -1.25 | 0.84 | 0.47 | | 0.65 |
| **Urgact** | -0.48 | 0.08 | | -1.10 | -0.48 | -0.30 | | 0.08 | 0.84 | 0.20 | | 0.62 | 0.18 | 0.22 | | 0.59 | 1.50 | 0.27 | | 1.59 | 0.18 | 0.13 | | 0.41 |
| **Modifcon** | -1.14 | 0.11 | | -0.47 |  | -0.29 | | 0.53 | 0.84 | 0.13 | | -0.20 | -0.48 | 0.09 | | -0.51 | -1.14 | 0.02 | | -1.36 | 0.18 | 0.34 | | 0.40 |
| **Adjustcon** | 0.18 | 0.03 | | -0.26 | 0.84 | 0.14 | | 1.07 | 0.84 | -0.03 | | -0.78 | 0.18 | 0.01 | | -0.46 | -0.48 | 0.04 | | -1.13 | 0.84 | -0.07 | | -0.43 |
| **Corr.** | 0.33 | | 0.58 | | 0.69 | | 0.62 | | 0.70 | | 0.77 | | 0.38 | | 0.59 | | 0.62 | | 0.46 | | 0.46 | | 0.69 | |
|  | 0.52 | | | | 0.58 | | | | 0.68 | | | | 0.39 | | | | 0.57 | | | | 0.50 | | | |

| **Emotion** | | | | | | | | | | | | | | | | | | | | | | | | | | | |
| --- | --- | --- | --- | --- | --- | --- | --- | --- | --- | --- | --- | --- | --- | --- | --- | --- | --- | --- | --- | --- | --- | --- | --- | --- | --- | --- | --- |
| **Shame** | | | | **Contempt** | | | | **Guilt** | | | | **Disgust** | | | | **Pleasure** | | | | **Despair** | | | | **Pride** | | | |
| **THEO** | **GEA** | | **GRID** | **THEO** | **GEA** | | **GRID** | **THEO** | **GEA** | | **GRID** | **THEO** | **GEA** | | **GRID** | **THEO** | **GEA** | | **GRID** | **THEO** | **GEA** | | **GRID** | **THEO** | **GEA** | | **GRID** |
|  | -0.46 | | -0.61 |  | -0.56 | | -0.76 |  | -0.46 | | -0.40 | -1.14 | -0.54 | | -0.70 | 0.84 | 1.38 | | 1.58 |  | -0.59 | | -0.88 |  | 1.38 | | 1.23 |
|  | 0.46 | | 0.55 |  | 0.40 | | 0.63 |  | 0.45 | | 0.34 | 1.50 | 0.54 | | 0.97 | -1.14 | -1.30 | | -1.70 |  | 0.60 | | 0.79 |  | -1.31 | | -1.20 |
| -0.48 | 0.09 | | 0.65 | -1.14 | -0.05 | | -1.50 |  | 0.19 | | -0.72 |  | -0.04 | | 0.31 | -0.48 | -0.24 | | 0.29 | 0.84 | 0.16 | | -0.50 |  | -0.51 | | -0.52 |
|  | 0.03 | | 0.13 | 0.84 | -0.12 | | -0.93 |  | 0.10 | | -0.39 | -0.48 | -0.03 | | 0.31 | 0.18 | 0.17 | | -0.28 | -1.14 | -0.05 | | -0.24 |  | 0.32 | | -0.86 |
|  | 0.20 | | -0.66 | 0.84 | 0.11 | | -0.07 |  | -0.07 | | -0.34 | -0.48 | -0.09 | | -1.06 |  | 0.12 | | 1.48 | -1.14 | -0.07 | | -0.67 |  | 0.04 | | 0.76 |
| 0.84 | -0.06 | | -0.93 | -0.48 | -0.35 | | -1.07 | 0.84 | 0.10 | | -0.90 | -0.48 | -0.23 | | -1.13 | 0.18 | -0.28 | | 0.92 | 1.50 | 0.22 | | -0.44 | 0.84 | 0.28 | | 1.45 |
| 0.18 | -0.15 | |  | -1.14 | -0.29 | |  | -1.14 | -0.20 | |  | -0.48 | -0.52 | |  | -1.14 | 0.56 | |  |  | -0.26 | |  | 0.84 | 0.76 | |  |
| 0.18 | 0.37 | | 0.99 | 0.84 | 0.49 | | 1.39 | 1.50 | 0.18 | | 1.21 | 0.84 | 0.63 | | 1.16 | -1.14 | -0.29 | | -1.27 |  | 0.10 | | 0.27 | -1.14 | -0.42 | | -1.42 |
| -1.14 | -0.15 | | -0.38 | -1.14 | -0.36 | | -1.06 | -1.14 | 0.03 | | -0.90 |  | -0.26 | | 0.02 | -0.48 | 0.07 | | 0.92 | 1.50 | -0.05 | | -0.38 | -1.14 | -0.20 | | -0.17 |
| 1.50 | 0.50 | | 0.69 | -1.14 | -0.29 | | -0.63 | 1.50 | 0.35 | | 1.25 | -1.14 | -0.31 | | -1.36 |  | 0.36 | | 1.05 | -1.14 | -0.16 | | -0.44 | 1.50 | 0.61 | | 1.71 |
| 0.84 | -0.08 | |  | -1.14 | -0.28 | |  | 1.50 | 0.04 | |  | -1.14 | -0.23 | |  |  | 0.69 | |  | -1.14 | -0.34 | |  | 1.50 | 0.93 | |  |
| -1.14 | -0.03 | | 0.71 |  | -0.15 | | 0.88 | -1.14 | -0.11 | | 0.56 |  | -0.23 | | 1.04 | 1.50 | 0.42 | | -1.41 |  | -0.19 | | 0.69 | 1.50 | 0.65 | | -1.24 |
| -1.14 | -0.07 | | -1.27 | 1.50 | 0.43 | | 0.71 | -1.14 | -0.25 | | -2.85 |  | 0.27 | | 0.70 | 0.84 | 0.11 | | 0.51 |  | 0.10 | | -0.53 | -1.14 | -0.07 | | -1.53 |
| -1.14 | -0.08 | | -0.71 | 1.50 | 0.39 | | 0.37 | -1.14 | -0.27 | | 0.03 |  | 0.33 | | -0.20 | 0.84 | 0.28 | | 1.69 |  | -0.05 | | -1.15 | -1.14 | 0.06 | | 1.06 |
| 1.50 | 0.00 | |  | 0.84 | 0.30 | |  | 1.50 | 0.03 | |  | 1.50 | -0.11 | |  | 1.50 | 0.22 | |  | 1.50 | -0.05 | |  | 1.50 | 0.19 | |  |
|  | -0.13 | | -0.54 | 0.84 | 0.16 | | 0.46 |  | -0.09 | | 0.46 | -0.48 | -0.16 | | -0.73 | 0.84 | 0.33 | | 1.63 | -1.14 | -0.14 | | -0.78 |  | 0.32 | | 0.79 |
| 0.84 | -0.03 | |  | 0.18 | 0.14 | |  | 0.84 | -0.04 | |  | -1.14 | -0.04 | |  | 0.84 | 0.23 | |  | 0.18 | -0.14 | |  | 0.18 | 0.21 | |  |
| -1.14 | -0.01 | |  | -1.14 | -0.10 | |  | -0.48 | -0.05 | |  | -1.14 | 0.07 | |  | -1.14 | 0.04 | |  | -1.14 | 0.06 | |  | -1.14 | -0.14 | |  |
|  | -0.38 | | -0.61 |  | -0.42 | | -0.70 | 0.18 | -0.36 | | -0.39 |  | -0.31 | | -0.74 | 0.84 | 1.02 | | 1.54 | -1.14 | -0.46 | | -0.82 | 0.84 | 1.17 | | 1.25 |
|  | 0.35 | | 0.64 |  | 0.4 | | 0.46 | -1.14 | 0.32 | | 0.30 |  | 0.29 | | 0.86 | -1.14 | -0.89 | | -1.49 | 1.50 | 0.52 | | 0.86 | -1.14 | -1.01 | | -1.15 |
|  | 0.21 | | 0.45 | 0.18 | 0.57 | | 0.95 | 0.18 | 0.11 | | -0.65 | -1.14 | 0.55 | | 0.94 | -1.14 | -0.73 | | -1.55 |  | 0.40 | | 0.63 | -1.14 | -0.79 | | -1.20 |
|  | 0.49 | | 0.83 | 0.84 | 0.45 | | 0.92 |  | 0.35 | | 2.13 |  | 0.37 | | -0.10 |  | -0.52 | | -0.37 | -1.14 | 0.16 | | -0.74 |  | -0.66 | | -0.43 |
| 0.84 | 0.06 | | -0.58 | -0.48 | 0.04 | | -0.92 | 0.18 | 0.16 | | -0.42 | 0.18 | 0.20 | | 0.31 | -1.14 | -0.45 | | 0.18 | 1.50 | 0.15 | | -0.46 | -1.14 | -0.36 | | -0.19 |
|  | 0.30 | | 0.59 | 0.18 | 0.20 | | 0.23 |  | 0.16 | | 1.42 |  | 0.12 | | -0.25 |  | -0.28 | | 1.17 | -1.14 | 0.11 | | -1.26 | 0.84 | -0.32 | | 0.44 |
| 0.18 | -0.02 | | -0.52 | 0.84 | -0.04 | | -0.53 | 0.18 | 0.08 | | -0.75 | 0.84 | -0.11 | | -0.80 | 1.50 | -0.02 | | 1.29 | -1.14 | -0.21 | | -1.50 | 0.84 | 0.02 | | 0.70 |
| 0.39 | | 0.72 | | 0.75 | | 0.78 | | 0.44 | | 0.47 | | 0.41 | | 0.68 | | 0.60 | | 0.66 | | 0.55 | | 0.59 | | 0.74 | | 0.62 | |
| 0.17 | | | | 0.68 | | | | 0.43 | | | | 0.47 | | | | 0.61 | | | | 0.58 | | | | 0.55 | | | |

Table S2

| **Appraisal** | **Code** | **Weight** | **Question** |
| --- | --- | --- | --- |
| Un/pleasantness | Un/pleas | 1 | How would you evaluate this type of event in general, independent of your specific needs and desires in the situation you reported above? |
| Suddeness | Sudden | 3 | The event happened very suddenly and abruptly? |
| Predictability | Predict | 1 | You could have predicted the occurrence of the event? |
| Familiarity / Novelty | Famil | 1 | You are familiar with this type of event? |
| Goal / Need importance | Impcons | 5 | The event would have very important consequences for you? |
| Moral acceptability | Moralacc | 7.5 | The actions that produced the event were morally and ethically acceptable? |
| Norm violation | Violnorm | 3.5 | The actions that produced the event violated laws or social norms? |
| Agency: chance | Chance | 1 | Chance, special circumstances, or natural forces? |
| Agency: self | Ownbehav | 2 | Your own behavior? |
| Intentionality: self | Intent | 1 | — If so, did you cause the event intentionally? |
| Image compatibility | Consimag | 10 | — If so, was your behavior consistent with the image you have of yourself? |
| Agency: other | Othbehav | 3 | The behavior of one or more other person(s)? |
| Intentionality: other | Othint | 3 | — If so, did (this) these other person(s) cause the event intentionally? |
| Consequences: known | Consfelt | 4 | At the time of experiencing the emotion, did you think that real or potential consequences of the event… had already been felt by you or were completely predictable? |
| Consequences: expectedness | Consexp | 1.5 | At the time of experiencing the emotion, did you think that real or potential consequences of the event… had been expected to occur at that time and in that specific form? |
| Consequences: near future | Conearfu | 1 | At the time of experiencing the emotion, did you think that real or potential consequences of the event… could be clearly envisaged and might occur in the near future with a fairly high probability? |
| Consequences: far future | Cofarfu | 4 | At the time of experiencing the emotion, did you think that real or potential consequences of the event… were somewhat unpredictable but might occur in the distant future (with uncertain probability)? |
| Goal conduciveness | Posoutc | 4 | At the time of experiencing the emotion, did you think that real or potential consequences of the event… did or would bring about positive, desirable outcomes for you (e.g., helping you to reach a goal, giving pleasure, or terminating an unpleasant situation)? |
| Goal obstructiveness | Negoutc | 4 | At the time of experiencing the emotion, did you think that real or potential consequences of the event… did or would bring about negative, undesirable outcomes for you (e.g., preventing you from reaching a goal or satisfying a need, resulting in bodily harm, or producing unpleasant feelings)? |
| Injustice | Unjust | 4 | At the time of experiencing the emotion, did you think that real or potential consequences of the event… were or would be unjust or unfair? |
| Coping | Avoidabl | 1.5 | At the time of experiencing the emotion, did you think that real or potential consequences of the event… could have been or could still be avoided or modified by appropriate human action? |
| Urgency | Urgact | 5 | That it was urgent to act immediately? |
| Power | Modifcon | 3 | That you would be able to avoid the consequences or modify them to your advantage (through your own power or helped by others)? |
| Adjustment | Adjustcon | 3 | That you could live with, and adjust to, the consequences of the event that could not possibly be avoided or modified? |

Figure S1


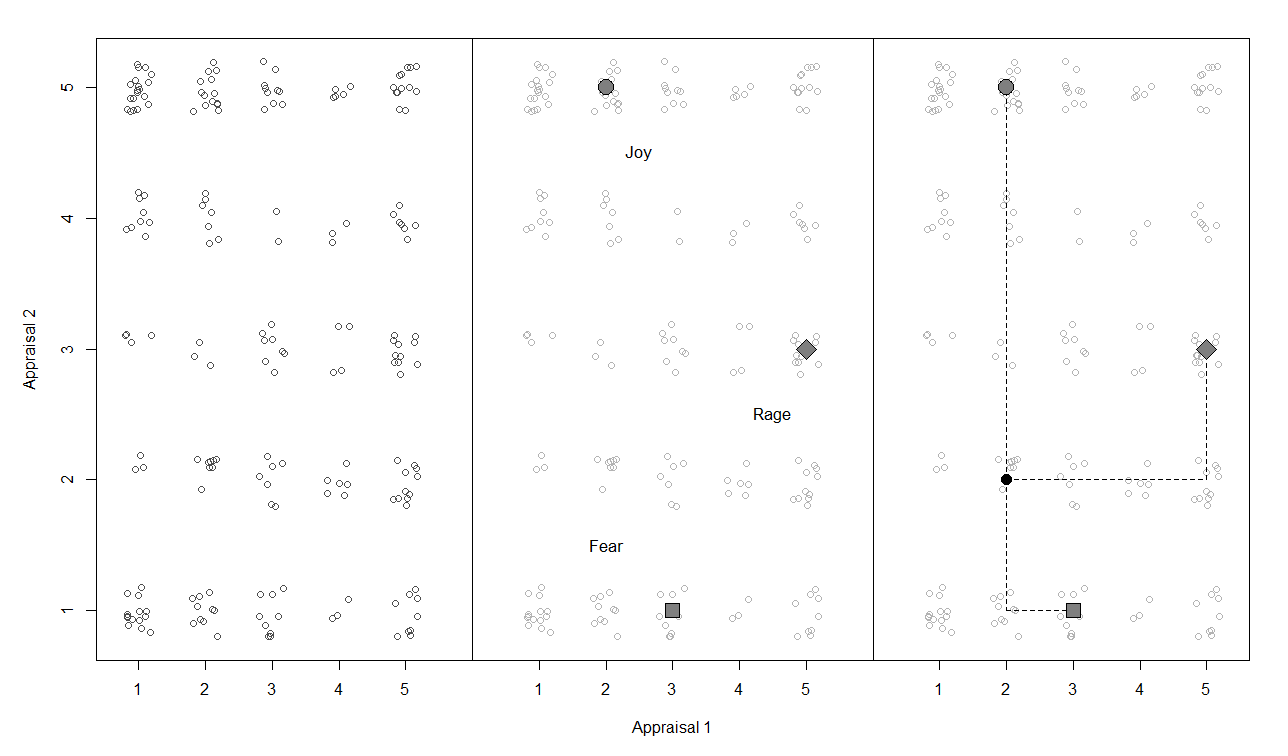

Supplement: File S1 — Supporting Information. Figure S1, Prototype matching with Manhattan distance for two-dimensional artificial appraisal data. User-observed appraisal input is depicted as data clouds in the 5 by 5 appraisal space. Theoretical emotion centroids for joy (circle), rage (diamond) and fear (square) are situated in this appraisal space. For a given user’s appraisal input (black dot), the GEA system calculates the Manhattan distance to each emotion centroid. The emotion with the shortest distance is outputted as the GEA’s prediction, in this case fear. Table S1, Standardized appraisal profiles for the 13 Geneva Emotion Analyst emotions. Displayed are the theoretically predicted profiles (THEO), the empirically found profiles for the GEA data (GEA), and the empirically found profiles for the GRID data (GRID). Pearson correlations between each pair of columns are displayed on the bottom row. Empty cells indicate that the corresponding appraisal item was not present for the respective profile. Refer to Table S2 for appraisal variable names. Table S2, List of appraisal questions in the GEA questionnaire, including appraisal check name, data abbreviation, and weight used in the adjustment of the distance functions. (DOCX) [file pone.0058166.s001.docx]
